# Vaccine Aphorisms

**Published:** 1846-04

**Authors:** 


17. *Vaccine Aphorisms.*—Some very excellent observations have recently appeared in the N. Hampshire Patriot, on the value of vaccination and the signs by which its perfect condition and protective influence may be known. The article was one that would have been creditable to any practitioner, and should, therefore, have had its place in a medical journal, where it would have been seen by five hundred practitioners where it is now noticed by one. The concluding aphorisms so entirely meet our individual views, that we have transferred them to our own pages—regretting, at the same time, that it is impossible to give credit for them to the close observer from whom they emanated, as he has concealed his name under the no-meaning signature of T.

1. A "scar" of the cowpox, having all the characteristic marks, and perfectly distinct, is not to be relied upon as an evidence of certain protection, unless proved by revaccination.

2. *Complete* vaccination forever secures an immunity to the individual from infection of the smallpox, varioloid, and from specific effects of cowpox.

3. In primary successful vaccinations, there is little evidence of the operation of the virus, before the fifth or 6th day, while in subsequent vaccinations, if the first be *complete*, there is speedy inflammation and itching, and the whole disappears at about the time the first should distinctly appear.

4. The most rigid scrutiny is requisite in the selection of matter, that it be collected at the right period of the vesicle, and from individuals of robust health, free from any cutaneous or other disorders, and of these conditions the physician is the only competent judge.

5. Cutaneous diseases (as perhaps visceral and other disorders,) seriously modify the character of the genuine vaccine affection, both as it regards the purity of the virus, for transferring the disease, and also the degree of security afforded the vaccinated, in consequence of impaired susceptibility.

6. *Re-vaccinate in all cases*, and repeat the operation so long as it specifically affects the system.

